# Effects of Dredging Induced Resuspension of Fine-Grain Sediment on Two Scleractinian Corals, *Montastraea cavernosa* and *Stephanocoenia intersepta*

**Published:** 2024-11-15

**Authors:** Cheryl Hankins, Keisha D. Bahr, Daphne White, Yung Jones, Adam Glahn, Wade Lehmann, Vladimir Kosmynin, William S. Fisher

**Affiliations:** 1 United States Environmental Protection Agency, Center for Environmental Measurement & Modeling, Gulf Breeze, USA; 2 Department of Marine and Coastal Environmental Science, Texas A & M University Corpus Christi, Corpus Christi, Texas USA; 3 United States Environmental Protection Agency, Office of Research and Development, ORISE Research Participation Program, Gulf Breeze, USA; 4 United States Environmental Protection Agency, Region 4, Water Division, Atlanta, USA; 5 Florida Department of Environmental Protection, Tallahassee, USA

**Keywords:** Dredging, Turbidity, Coral, Light, Photosynthetically active radiation

## Abstract

Dredging, the removal of sediments and rocks from an aquatic environment, is necessary to ensure that adequate coastal infrastructure is maintained for maritime shipping. However, the sediment plumes generated by dredging could have adverse impacts on coral reef ecosystems that are already facing numerous local and global stressors. This is especially true in areas where the predominant strata are aragonitic limestone which must be physically broken apart to be extracted, leading to very high concentrations of fine suspended sediment in the water column. To examine the role suspended sediment plays in stress, this study exposed two coral species (*Montastraea cavernosa and Stephanocoenia intersepta*) to fine-grain sediment ranging from 0 to 511.7 mg L^−1^ for 30 days. Sediment characteristics were analyzed and water quality parameters were monitored. Growth, mortality and three bleaching indicators were documented after 10 and 30 days. No mortality or tissue loss was observed in either species. No significant differences in growth were observed in the 10-day exposure; however, both species had significant declines in calcification at the 30-day exposure. Bleaching indicators did not appear to be affected by sediment exposure at day 10 or 30. Reduction in photosynthetically active radiation was highly correlated with both turbidity metrics of NTU units and Total Suspended Solids. These data indicate that sediment may minimally impact coral in short-term exposures but can affect growth in longer-term exposures in multi-year dredging projects in sensitive tropical and sub-tropical environments.

## INTRODUCTION

Coral reefs of the Anthropocene face numerous stressors. Nearly every geographic region with coral reefs has seen substantial declines in coral communities [1–5]. Corals are subjected to global stressors (i.e., ocean acidification and elevated temperature) and experience local pressures such as overfishing, diseases and coastal development [5–7]. The future of coral reefs relies on understanding these stressors and how to better mitigate their interactive effects [8].

Nearly 13% of the global population (approximately 1 billion people) lives within 100 km of a coral reef, with continued population growth near coral reefs outpacing global averages [9]. As populations increase, so does the need for coastal development. Often, this development relies on nearshore dredging to remove material from aquatic environments such as port expansions for the maritime industry. According to the Organization for Economic and Co-operation and Development, approximately 90% of international trade is conducted through maritime shipping [10]. The expansion of the Panama Canal in 2016 allowed the passage of longer and wider vessels to access ports across the world [11,12]. Many ports have updated their infrastructure to accommodate these larger, Neo-Panamax ships which has required dredging to deepen and widen channels [13]. However, upgrades in coastal infrastructure can also impact vulnerable coral reef communities [14].

The earliest records of dredging impacts on coral date from the 1970s [15,16]. Fine sediments (<63 μm) generated by dredge operations on adjacent reef and hardbottom habitats have numerous consequences for corals, including decreased recruitment, growth, species diversity and coral cover and increased bleaching, disease, tissue loss and mortality [14,17–20]. The causeeffect pathways associated with these sediment effects were reduced Photosynthetically Active Radiation (PAR) needed for zooxanthellae photosynthesis, increased turbidity which reduced PAR levels and sediment deposition resulting in coral burial, all of which are heavily influenced by grain size of the sediment [21–25]. The overall risk to coral may also depend on coral species and morphology, water currents and the intensity and duration of the water column impacts from dredging operations [17,26–28]. Although effects of sediment on coral have been documented, the relation of those effects to measurable water quality metrics is lacking. Currently, there are insufficient data linking suspended sediment to effects on coral for use in generating protective water quality standards. According to the Western Australian Marine Science Institution thresholds need to be established for factors affecting water quality parameters including light reduction, suspended sediment and sediment deposition [24,29–31].

The global increase in port expansions and other dredging activities illustrate the need for water quality metrics that can be used to protect coral. There is also a need for further studies on Atlantic corals since <20% of documented studies have been conducted on Atlantic species [31]. Responses to water quality perturbation by suspended sediment, as is found during dredging in tropical/sub-tropical regions, was monitored in this laboratory study using two Atlantic/Caribbean coral species commonly found in Florida and the Caribbean, *Montastraea cavernosa* and *Stephanocoenia intersepta*. Colonies were exposed for 10 and 30 days to low-nutrient, fine, calcareous sediment commonly released during dredging activities near reef environments. Coral growth, mortality and bleaching indicators including zooxanthellae density, chlorophyll-a and protein content were measured and multiple water quality parameters (turbidity, total suspended solids, photosynthetically active radiation) were monitored.

## MATERIALS AND METHODS

*Montastraea cavernosa* and *Stephanocoenia intersepta* colonies were obtained from Florida Keys National Marine Sanctuary Coral Nursery Program and Mote Marine Laboratory’s on-site coral nursery located near Looe Key (Permit number FKNMS-2017-151). Corals were shipped to the indoor Coral Research Facility at the U.S. Environmental Protection Agency’s Gulf Ecosystem Measurement and Modeling Division in Gulf Breeze, Florida and maintained for at least three months in recirculating culture systems (~1000 L). Culture systems were kept at a temperature of 26.0°C ± 1.0°C and salinity of 35.0 ± 0.3 ppt. Lighting was provided by metal halide lights on a 10.5:13.5 light:dark cycle.

To determine the amount of sediment needed for turbidity treatments, preliminary studies were conducted by placing the coral skeletons of each species into four experimental containers with pumps for circulation. Sediment was added to the containers until the desired turbidity was reached and then turbidity was measured twice a day. As sediment settled, turbidity levels declined to unacceptable levels. Stirring with a pump for ~20 sec twice per day resulted in pulsed sediment exposure. Stirring occurred in conjunction with morning and afternoon checks of the experimental system. Water changes and intermittent cleaning of the containers were also found necessary to keep sediment in suspension and maintain turbidity closer to target levels. On day 0, Total Suspended Solids (TSS) measurements (dry weight) were converted to wet weight to determine the amount of sediment needed to replenish the amount removed during water changes; turbidity and Photosynthetically Active Radiation (PAR), in addition to TSS, was also measured on day 0 for regression analysis. Preliminary studies showed that a pump placed approximately 15 cm from the bottom of a chamber with no coral had turbidity values that exceeded the 1000 Nephelometric Turbidity Unit (NTU) level of the meter for the highest treatment (511.7 mg L^−1^) described below. These values changed with placement of the pump and contents of the chambers.

Coral colonies used in the experiment were approximately 40 cm^2^. Parent colonies that were larger than 40 cm^2^ were cut at least two months before experimentation and held in culture systems. Cuts were designed to create a surface area of approximately 40 cm^2^ and to maintain as close to a natural morphology as possible. Test specimens were cut from 12 M. *cavernosa* colonies and 22 *S. intersepta* colonies. Two test colonies of each species were transferred into 24 plastic, circular 8 L containers with 7 L sea water and held for 7 days of acclimation in the experimental system prior to exposure. Each chamber had a pump that hung from a bracket for water continuous circulation (Supplementary Figure 1). Water flow from the experimental system through the chambers was on for the first day for 5 h after transfer but then flow was terminated during the final 6 days to acclimate the corals to a static system that underwent 25% water changes every 2 days. Corals were fed twice per week 15 min prior to a water change.

The protocol and system described above were sustained throughout 30 days of experimental treatment. Test colonies were sampled at day 10 to follow chronic marine sediment toxicity testing protocols for Ecological Risk Assessments and at day 30 to provide a longer exposure duration [32]. Six sediment treatments were tested in four replicate containers, each holding two test colonies of each species in 7 L seawater. Turbidity, in NTU units, was targeted as the parameter for selecting experimental treatments with nominal values of 0 (control), 25, 50, 100, 200 and 400 NTU which, as determined from the preliminary experiment, translated to dry weight values of 0, 5.4, 73.9, 142.1, 284.3 and 511.7 mg L^−1^, respectively. These sediment amounts were added to containers on day 0 and again (same amounts) on day 10 and day 20 when containers were emptied, cleaned to prevent excessive algae accumulation and 100% water exchanged. Sediment was added to the chambers as initial doses (days 0, 10 and 20) and aerated overnight in seawater prior to addition. Exposure duration was 10 days for half the colonies and 30 days for the other half.

### Sediment analysis

The sediment used for the study was collected with a shovel near an uninhabited island (Porpoise Key 24 ° 43’ 15.03” N, 81 ° 21’ 10.42” W) in the Florida Keys, Florida, USA to a sediment depth of 35 cm at a water depth of 1.0-1.2 m. Sediment was transported in five 20 L sealed buckets to the coral research facility in Gulf Breeze, Florida. Sediment was sieved to remove contents greater than 4 mm and then homogenized in a single holding container. The sediment grain size was determined by a laser diffraction particle size analyzer with Deionized (DI) water as a dispersant. Sediment was also tested for physical properties, i.e. water content (method 200.2), total organic content (United States Environmental Protection Agency (USEPA) Region 4 Standard Operating Procedure LSBPROC-065-R1), metals, semivolatile organic compounds and organopesticides, Polychlorinated Biphenyl (PCB) congeners and ratio of calcium carbonate (marine):terrigenous content (B & B Laboratories, Inc. Standard Operating Procedure 1005) [33–37].

### Water quality

Water quality parameters were all measured prior to experimental exposure and either weekly or bi-weekly thereafter to ensure accordance with culture condition parameters [38–40]. These included pH (Nature-based solutions (NBS); YSI^®^ Ecosence pH100), calcium (parts per million (ppm); Salifert^®^), alkalinity (Degrees of german carbonate hardness (dKH); Salifert^®^), magnesium (ppm; Salifert^®^), ammonia (mg L^−1^; HACH^®^), phosphate (mg L^−1^; HACH^®^) and nitrate (ppm; Salifert^®^). All water samples were extracted using a 60 mL syringe with a 0.20 μm syringe filter. Additionally, light intensity (W m^−2^) was measured with a Macam^®^ radiometer (Model UV203-3) before experimentation and after 100% water renewals. Turbidity (NTU) was measured (HACH^®^ 2100Q) three times per week with alternating measurements between pre- and post-stirs. All parameters were grouped into two exposure periods, days 0-10 or 0-30 and tested for normality using the Anderson-Darling test. If parametric, one-way Analysis of Variance (ANOVA) with a post-hoc Tukey test was performed. If nonparametric, a Kruskal Wallis was performed followed by Bonferroni’s multiple comparisons if a significant difference was detected (Minitab 19, Inc.). For comparisons among NTU, light and TSS, all were measured within an hour of a sediment addition prior to the addition of corals on day 0. Graphs of mean were generated in Sigma Plot 15, pairwise comparison graphs generated in Minitab 19, Inc.

### Tissue surface area and mortality

The live Tissue Surface Area (TSA) of each test colony was determined on days 0, 10 and 30. Test colonies were removed from chambers, gently rinsed to remove sediment and placed onto a turntable for easy rotation. A three-Dimensional (3D) scan was obtained by using an Artec^®^ Space Spider 3D scanner [41]. The scanner was held approximately 30 cm from the coral roughly at a 30° horizontal angle while the turntable was rotated 360°. The scanner was moved to an approximate 80° angle in the same scan and another rotation was completed. A scan took approximately two minutes during which the test colony was exposed to air. Using Artec^®^ Studio software, the tissue surface area of the colony was isolated and the surface area was calculated. The percent change of tissue surface area for days 10 and 30 was tested for normality using Anderson-Darling test. Separate one-way ANOVAs or Kruskal-Wallis tests were performed as appropriate for day 10 and day 30 data for each species (Minitab 19, Inc.). All graphs were generated in Sigma Plot 15.

### Calcification

Total alkalinity appears to be influenced by the addition of sediment as preliminary study measurements were not repeatable. Since alkalinity may be influenced by the addition of sediment, test colonies were moved to jars containing no sediment to measure total alkalinity for the calcification endpoint [42,43]. Total alkalinity was measured following the 10 and 30-day exposures. Test colonies (n=48 for each exposure period) were moved from experimental chambers and incubated in individual 3.7 L glass jars containing 2.3 L of seawater (Supplementary Figure 2). Jars contained air stones to supply water movement. Water samples from each jar were collected prior to coral transfer (time point 1) and after the coral resided in the jar for 24 h (time point 2). Total alkalinity was measured by open-celled titration [44]. Total alkalinity was used to calculate calcification based on the alkalinity anomaly principle, which assumes that for every one mole of calcium carbonate precipitated, total alkalinity decreases by two moles [45]. The following equation determined calcification:

$$\frac{\text{Δ}A(\text{T})}{2}=G$$

where, A(T) is total alkalinity (μmol kg^−1^) and G is calcification, i.e., calcium carbonate precipitated (μmol kg^−1^). Calcification data were normalized to the tissue surface area of the coral colony on the day they were sampled (day 10 or 30), tested for normality using the Anderson Darling test and analyzed using a one-way ANOVA with Tukey’s post-hoc test (Minitab 19, Inc.). If data did not meet the assumptions of normality, a Kruskal Wallis test was performed. All graphs were generated in Sigma Plot 15.

### Mortality

Mortality was observed daily and a record of date was recorded. A coral fragment was considered dead when there was 0% tissue remaining. Total mortality was calculated at day 10 and 30.

### Bleaching indicators

After total alkalinity water samples from time point 2 were collected, test colonies were removed from jars, wrapped in aluminum foil, placed in Whirl-Pak^®^ bags and immediately placed in a −80°C ultralow freezer. Coral samples were shipped to Texas A&M University-Corpus Christi on dry ice where coral tissue was removed using an airbrush (Paasche, D500SR) and a compressor that jets high-pressure Phosphate-Buffer Saline Solution (PBS) to create tissue slurry. The tissue slurry was collected in a plastic bag and transferred into a 50 mL Falcon tube up to 42 mL. Samples were homogenized with a Tissue Master homogenizing probe. The tissue slurry was centrifuged (3000 Rotations per Minute (rpm) for five minutes) to separate the zooxanthellae (i.e., algal pellet) from the coral (i.e., supernatant). Aliquots (1 mL) were withdrawn from the coral tissue slurry supernatant liquid for total protein analysis of the coral as described below. The remaining supernatant liquid was discarded, leaving only the algal pellet at the bottom of the 50 mL tube [46]. Next, 5 mL of PBS was added to the algal pellet and vortexed until fully mixed. Samples were aliquoted (1 mL each) into plastic vials and stored in a −20°C freezer until processing for zooxanthellae density, chlorophyll and protein concentrations.

Zooxanthellae were counted from the frozen samples using a hemacytometer at 10x magnification on a Leica DM500 compound microscope [47,48]. All samples were counted twice, averaged and then standardized to the coral surface area at which they were sampled (day 10 or 30) ensuring that changes in density would not be conflated with changes in live tissue surface area. Chlorophyll concentrations were measured and calculated using methods from Jeffrey and Humphrey, Putnam and Edmunds [49,50]. Samples were removed from the freezer, thawed and centrifuged to remove PBS. Acetone (90%) was added to each sample, vortexed and kept in the dark at −20°C for 24 h. Samples were analyzed using a spectrophotometer at wavelengths for chlorophyll a (chl-a) (630 nm) and chlorophyll c_2_ (chl-c) (663 nm).

Total protein for the coral was analyzed using protein spectrophotometry methods [46]. Aliquots (1 mL) of the coral slurry supernatant, described above, were thawed to room temperature and read at wavelengths 235 nm and 280 nm. To ensure that the protein concentration fell within the desired range for each wavelength (0.1-1.0), the samples were diluted with PBS as needed. All measurements were standardized to the coral tissue surface area on the day on which they were sampled and to the dilutions used throughout processing and analysis. Coral skeletal density was calculated (measured mass divided by volume) using the water displacement method for each test fragment [51]. Zooxanthellae density and chlorophyll and protein concentration data for days 10 and 30 were tested for normality using Anderson-Darling test. Separate one-way ANOVAs or Kruskal-Wallis tests were performed as appropriate for day 10 and day 30 data for each species (Minitab 19, Inc.). All graphs were generated in Sigma Plot 15.

## RESULTS

### Sediment analysis

The grain size composition of the sediment used in these experiments was 2% clay (0-4 μm), 52% silt (5-63 μm) and 46% sand (64-2000 μm). Sediment consisted of an 82% calcium carbonate and a 6% terrigenous fraction with 38% solids (62% water content). The average total organic carbon was 56.7 g kg^−1^ (Standard Deviation=15.3). Aluminum and iron were metals with the highest concentrations of 690 and 670 mg kg^−1^ dry weight, respectively. All other metals were at concentrations <5.0 mg kg^−1^ dry weight. No semivolatile organic compounds, PCB congeners, or organochloride pesticides were detected.

### Water quality

There were no significant differences (p<0.05) among treatment groups for pH, calcium, nitrate, ammonia, or phosphates for either days 0-10 or days 0-30 and all were within normal culture conditions. Alkalinity levels generally increased from the control to the 511.7 treatment for both days 0-10 and 0-30 (Days 0-10: ANOVA, F=4.40, p=0.009, days 0-30: Kruskal Wallis, H=12.00, p=0.035) (Supplementary Figures 3 and 4). The 511.7 treatment was significantly different from all other treatments for each exposure period. All alkalinity levels were below the recommended, minimum level for culture, 7.0 dKH (days 0-10: 5.1 dKH-6.3 dKH, days 0-30: 5.6 dKH-6.9 dKH) (Supplementary Figure 3). Magnesium levels were different most notably between the control and 142.1 mg L^−1^ treatment for days 0-10 with no significant differences for days 0-30 (Supplementary Figure 4).

Mean turbidity values of each treatment between exposure periods were different, though not statistically. Taking a conservative approach, measurements for water quality, tissue surface area, calcification and bleaching indices were analyzed separately for days 0-10 (acute exposure) and days 0-30 (chronic exposure). Mean turbidity increased from the lowest sediment treatment to the highest and was also significantly different for both exposure periods between the controls and all other treatment comparisons (days 0-10: Kruskal Wallis, H=70.04, p=0.00, days 0-30: H=257.69, p=0.00; Bonferroni, comparisons=15, α=0.2, Z=2.475 and days 0-30: H=247.72, p=0.00; Bonferroni, comparisons=15, ɑ=0.013, Z=2.475), though not all treatments were significantly different from each other (Table 1) and (Supplementary Figures 5A and 5B). Pre and post-stir turbidity values varied greatly among each treatment, with reductions between 73.1% and 93.2% from initial measurements (Supplementary Table 1).

**Figure 1:**
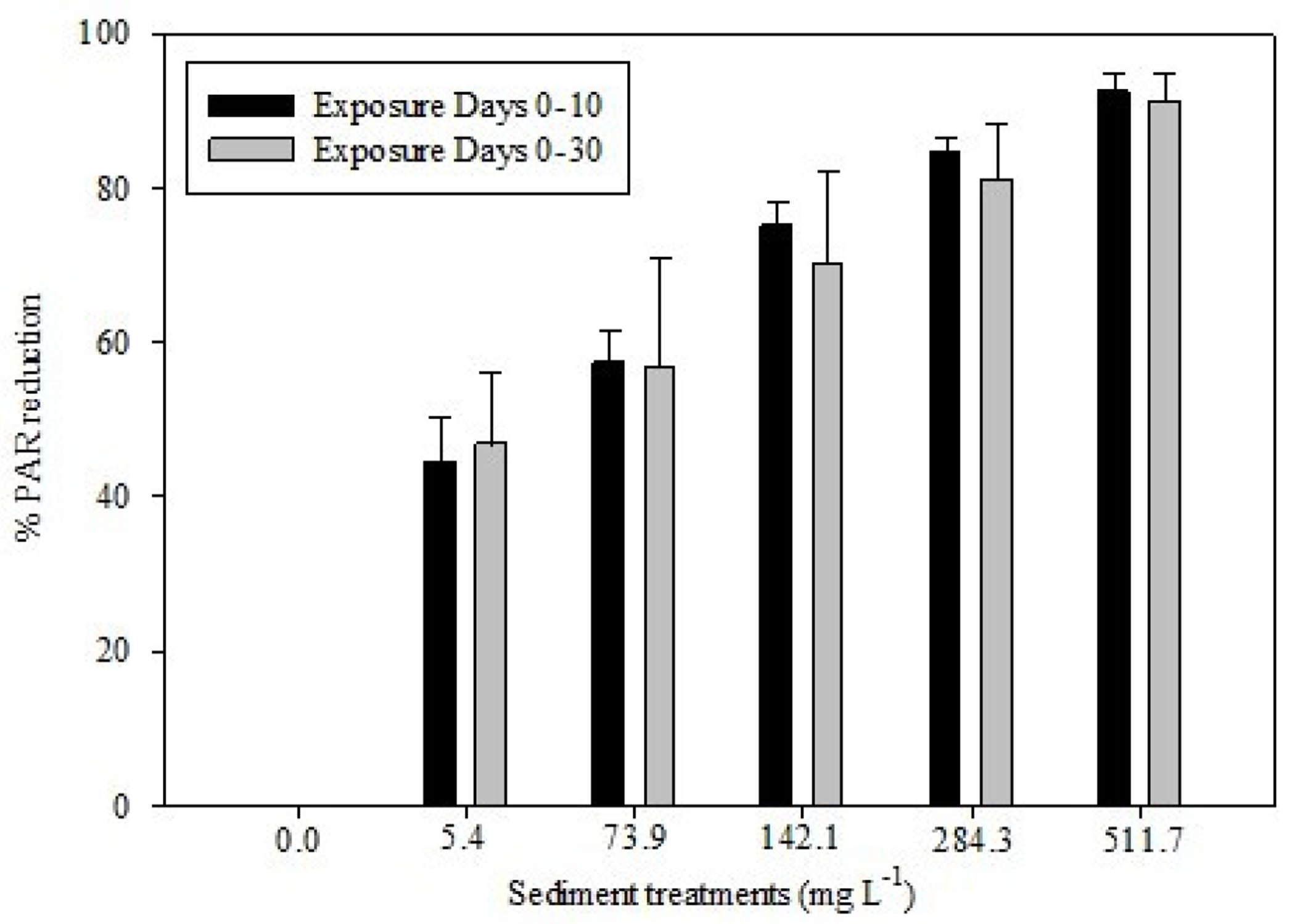
Mean percent reduction of Photosynthetically Active Radiation (PAR) for exposure days 0-10 and 0-30 (error bars represent standard deviation of the mean response for each treatment).

Photosynthetically Active Radiation (PAR) decreased with increasing sediment treatment, data is reported as mean percent PAR reduction (Figure 1). Significant differences among treatments were detected in PAR at both days 0-10 (Kruskal Wallis, H=22.40, p=0.00) and days 0-30 (Kruskal Wallis, H=104.65, p=0.00). Pairwise comparisons showed differences between treatment groups during days 0-10 and 0-30 (Supplementary Figures 6A and 6B).

**Figure 2:**
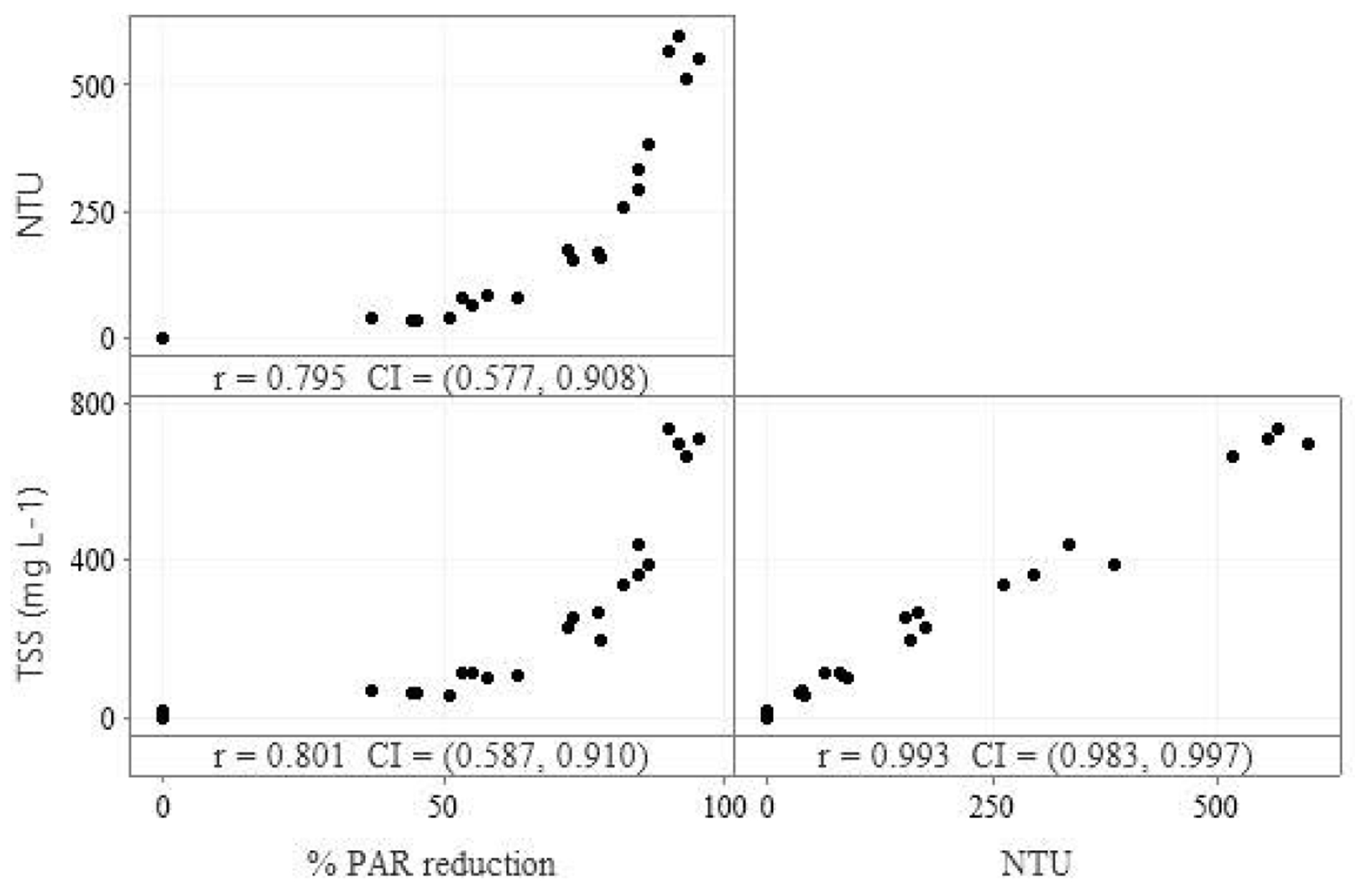
Pairwise linear regressions of sediment-influenced water quality parameters, reduction of Photosynthetically Active Radiation (PAR), turbidity and Total Suspended Solids (TSS). **Note:** All parameters were measured on day 0.

Of the 3 pairwise regressions for sediment-influenced water quality measurements (NTU, PAR and TSS), all exhibited strong correlation with each other (r>0.700) (Figure 2). Total Suspended Solids and NTU had the highest coefficient at r=0.993 with NTU and PAR reduction having the lowest at r=0.795.

### Tissue surface area and mortality

**Figure 3:**
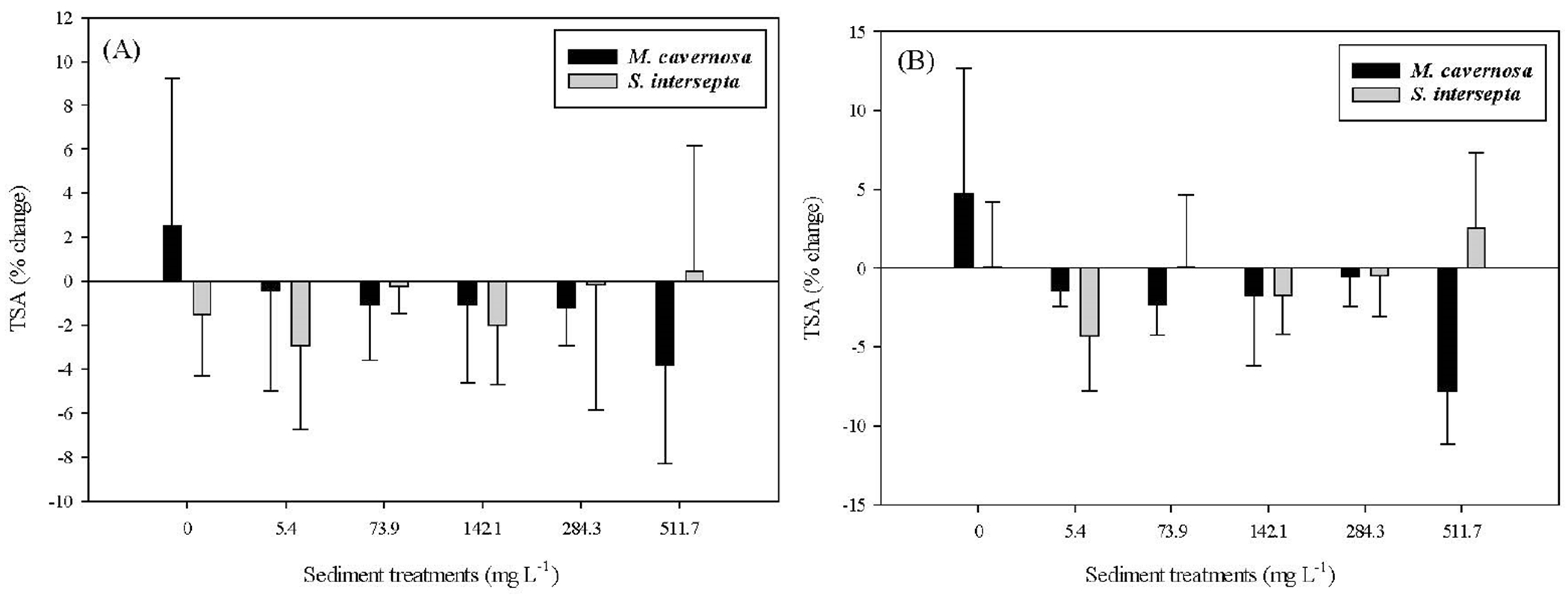
Mean percent tissue surface area change for *Montastraea cavernosa* and *Stephanocoenia intersepta*. **Note:** A) At day 10; B) At day 30; Error bars represent standard deviation of the mean response of species for each treatment.

By day 10, M. *cavernosa* Tissue Surface Area (TSA) declined with increasing sediment treatment though no significant differences (Kruskal Wallis, df=5, H=7.47, p=019) were detected (A). *Stephanocoenia intersepta* showed positive tissue growth at the highest treatment only, in all other treatments there was tissue loss though no significant differences occurred (Kruskal Wallis, df=5, H=5.17, p=0.40) (Figure 3A). By day 30, the *M. cavernosa* control treatment was the only group to have increased TSA, whereas the highest (511.7) treatment had the most tissue loss; however, no significant effects (p<0.05) were observed (Kruskal Wallis, df=5, H=10.63, p=0.06) (Figure 3A). The control and the 511.7 treatments in *S. intersepta* were the only groups to have gained tissue, with 511.7 having the highest tissue growth but again no significant differences were detected (ANOVA: df=5, F=1.23, p=0.34) (Figure 3B).

### Calcification

**Figure 4:**
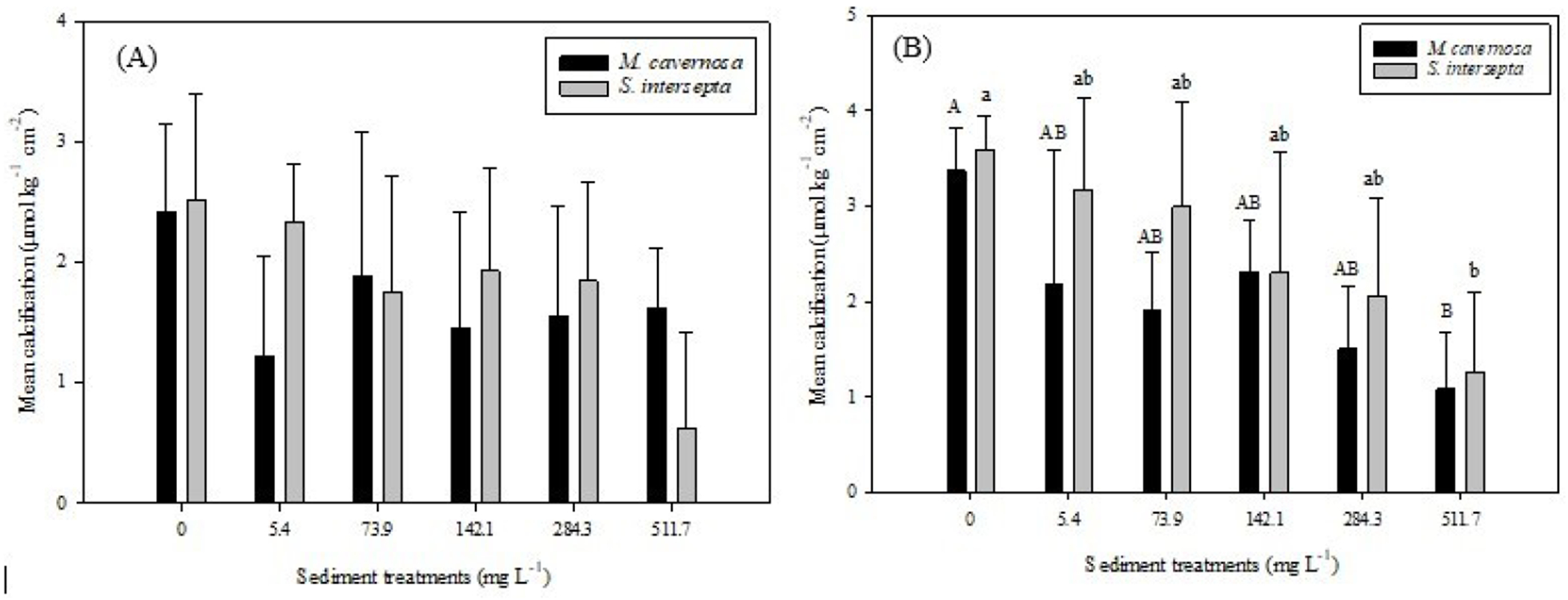
Mean calcification normalized to tissue surface area for *Montastraea cavernosa* and *Stephanocoenia intersepta*. **Note:** A) At day 10; B) At day 30; Error bars represent standard deviation of the mean response of species for each treatment. Capital letters indicate significant difference between treatment groups for *M. cavernosa* (Turkey’s post-hoc). Lower case letters indicate significant difference between treatment groups for *S. intersepta* (Turkey’s post-hoc).

At day 10, calcification was lower in all treatments compared to the controls, although no significant differences (p<0.05) were detected in for either species (ANOVA, *M. cavernosa*: df=5, F=0.76, p=0.59 and *S. intersepta*: df=5, F=2.57, p=0.06) (Figure 4). At day 30, calcification declined with increasing sediment treatment for both species except for the 142.1 treatment in *M. cavernosa* (Figure 4A). Significant differences in both species were observed at day 30 with differences seen between the control and 511.7 mg L^−1^ treatments (ANOVA, *M. cavernosa*: df=5, F=3.07, p=0.04 and *S. intersepta*: df=5, F=3.16, p=0.03) (Figure 4B).

### Mortality

No mortality was observed throughout the 30-day exposure.

### Bleaching indicators

Zooxanthellae density normalized to TSA, at days 10 and 30 were not significantly different for either *M. cavernosa* (ANOVA, Day 10: df=5, F=0.83, p=0.55 and Day 30: df=5, F=1.44, p=0.26) or *S. intersepta* (Kruskal-Wallis: Day 10: df=5, H=2.87, p=0.72 and Day 30: df=5, H=5.45, p=0.36) (Supplementary Figures 7 and 8). *Montastrea cavernosa* had lower mean zooxanthellae (zoox) densities across all treatments compared to *S. intersepta* at day 10 (87940 zoox per mm^−2^ SD=50668 and 200542 zoox per mm^−2^ SD=119719, respectively) and at day 30 (127355 zoox per mm^−2^ SD=45524 and 210594 zoox per mm^−2^ SD=122128). The highest mean density at day 10 was in the 284.3 treatment (126864 zoox per mm^−2^ SD=107031) and the lowest in the 5.4 treatment (62213 zoox per mm^−2^ SD=20197) (Supplementary Figure 7). At day 30, *M. cavernosa* had the highest density in the 511.7 treatment (177883 zoox per mm^−2^ SD=52628) and the lowest zooxanthellae density in the 5.4 treatment (106283 zoox per mm^−2^ SD=61908) (Supplementary Figure 8). *Stephanocoenia intersepta* at day 10 had the highest mean zooxanthellae density in the 142.1 treatment (303603 zoox per mm^−2^ SD=186342) and the lowest mean in 73.9 treatment (134902 zoox per mm^−2^ SD=39065) (Supplementary Figure 7). At day 30, the highest zooxanthellae density was in the 5.4 treatment (272614 zoox per mm^−2^ SD=138560) and the lowest density in the controls (108346 zoox per mm^−2^ SD=41084) (Supplementary Figure 8).

Chlorophyll-a concentration, normalized to TSA, was not significantly (p<0.05) different in any treatments for either *M. cavernosa* or *S. intersepta* at day 10 (ANOVA: df=5, F=0.47, p=0.80 and df=5, F=0.26, p=0.93, respectively) (Supplementary Figure 9). At day 30, there was no significant difference in *M. cavernosa* (Kruskal-Wallis: H=9.36, p=0.10) or *S. intersepta* (ANOVA: df=5, F=1.02, p=0.48) (Supplementary Figure 10).

Protein concentration, normalized to TSA, at day 10 was not significantly different (p<0.05) for *M. cavernosa* (Kruskal-Wallis: df=5, H=4.16, p=0.53) or *S. intersepta* (Kruskal-Wallis: df=5, H=4.37, p=0.50); nor was there a difference at day 30 for *M. cavernosa* (Kruskal-Wallis: df=5, H=10.25, p=0.07) or *S. intersepta* (ANOVA: df=5, F=0.30, p=0.91) (Supplementary Figures 11 and 12).

## DISCUSSION

The potential for direct effects of fine dredged sediments on corals is evaluated through controlled laboratory exposures including systematic water quality monitoring. Comparisons of select water quality parameters with coral growth responses and bleaching indicators provides insight into threshold values best suited to protect against effects to coral health during dredge operations. Specifically, this study documented PAR and two turbidity parameters, NTU and TSS, in relation to the responses of two stony coral species, *Montastraea cavernosa* and *Stephanocoenia intersepta*. Results indicate that neither of these two species had significant changes in bleaching indicators to sediment in the 10 days or 30-days exposures. Though no mortality or significant tissue loss was observed, calcification decreased in both *M. cavernosa* and *S. intersepta* at the end of day 30 in the highest sediment treatment. Generally, there was an upward trend in total alkalinity as sediment concentrations increased implying less calcification.

Standard sediment toxicity tests used for derivation of threshold values often entail a 10-day exposure to observe the growth and mortality of exposed organisms [32]. Due to the slow-growing nature of scleractinian coral, ten days may not be an adequate length of time to detect these responses. The lack of responses in *M. cavernosa* and *S. intersepta* at day 10 in this study suggests that acute coral duration exposures last more than 10 days. Additionally, suspended sediment has been noted to take 10 times longer to present tissue mortality than deposited sediment, which can bury the tissue and may provide an explanation for the absence of tissue mortality in this study [31]. The turbidity treatments selected represent the high to low gradient seen in active operations. The highest turbidity levels seen at the source of dredging are 500 mg L^−1^ and 400 NTU representing our highest targeted NTU treatment of 400 NTU (or 511.47 mg L^−1^). Low turbidity measurements occurring in an active dredging operation include a monthly average of 25 NTU and 10-80 mg L^−1^ each representing the lower end the sediment treatments used in this study [52,53].

The sediment collected for this study was chosen because of its similar characteristics to dredged material. The sea floor around coral reefs habitat consists largely of calcium carbonate material (aragonite); during the dredging process large amounts of silt and colloidal particles are generated, especially in cases where physical manipulation of underlying limestones is required. Thus, when dredging around coral reefs, the dredged material generally consists of fine, calcium carbonate sediments [14,24]. The sand content of the collected sediment was close to the fraction seen in Australian offshore environments after dredging activity [24]. Though silts (very fine-grain sizes) are a characteristic of sediment derived from terrigenous sources, the high calcium carbonate to terrigenous ratio of our sediment indicates it was primarily marine-derived [54,55].

The nominal turbidity levels could have been used for treatment classifications; however, the difference in mean turbidity measured between days 0-10 and days 0-30 was concerning. Even though no significant differences were detected between treatments of each exposure period, the low tolerances of some scleractinian corals were considered, therefore, we did not want to presume exposures had similar treatment conditions. The variability in turbidity seen in our exposures was partly due to biological conditions within the chambers. Algae would colonize the surfaces of the chambers within 48 h which attracted the fine sediment to aggregate on the surfaces, a condition seen in other laboratory experiments [56]. Additionally, there was some settling of the coarser grains at the bottom of the chambers. The higher turbidity observed during the 30-day exposure could also be a result of more open area in the chamber as there was half the number of colonies in each chamber after day 10. Ultimately, the variability seen in the experimental chambers may not be too unlike what was observed in dredging operations near a reef, where temporal variability of sediment flushing was influenced by tidal cycles and may be considered as pulsed events [24,30].

Many U.S. states, including the State of Florida currently assesses turbidity in NTU, thus making it ideal candidate for defining water quality criteria during dredging operations. Our comparative measurements indicate that NTU and TSS are strongly correlated implying that either parameter provides similar information of water clarity. Additionally, both turbidity units are correlated with the decrease in light. Publicly available data on water quality parameters during dredging operations is limited, however, our results support data [24,52]. whereby, as turbidity increases, PAR decreases. Similar correlations among water quality parameters have been noted in reef habitats after flood events and dredging operations [55].

Few studies have investigated the effects of suspended sediment on *M. cavernosa* and *S. intersepta*. However, the results of this study support other studies showing that both species are relatively tolerant to suspended sediment [57,58]. Rice and Hunter showed that in a 10-day exposure, growth in *S. intersepta* was not affected at 165 mg L^−1^, a dose slightly higher than the 142.1 mg L^−1^ treatment used in this study [57]. Likewise, *M. cavernosa* percent live tissue was not affected by a 72 h exposure, nor were there any signs of oxidative stress. However, photosynthetic efficiency was impacted by sediment at a concentration of 1047 mg cm^−2^, a value ten times higher than the highest treatment in this study (80 mg cm^−2^) [58]. Neither of these studies quantified additional sediment parameters or offered water quality measurements; it is only with the sediment measurements reported in this study that comparisons can be made among studies, thus emphasizing the need for reporting of both sediment and water quality parameters [31].

Dredging activity can elevate suspended sediment levels which can significantly reduce light availability underwater. Although corals are equipped to remain tolerant to changes in light availability, algal symbionts (zooxanthellae) sometimes respond to lower light conditions by decreasing densities (bleaching), which can lead to coral mortality [59,60]. In this study, none of the bleaching indicators were significant at the highest sediment concentration in the 10 or 30-day exposures. With both *M. cavernosa* and *S. intersepta* having relatively slow growth rates, especially compared to fastgrowing Acroporids, physiological impacts may take longer than 30 days for detection. At the 10-day exposure, chl-A and protein concentrations in *M. cavernosa* had high p-values (p>0.5) but at the end of the 30-day exposure p-values dropped <0.1 indicating a possible developing trend. Tolerant species, like *Stephanocoenia intersepta* have been shown to adapt in low light conditions by not decreasing symbiont densities, likely producing more chl to compensate for the reduced response [61]. However, less tolerant species that are highly abundant, like Montastrea cavernosa, can be more susceptible to synergized stressors during low light conditions that can cause a decrease in symbiont density [61,62]. Therefore, further information is necessary for dredging management to determine both acute and chronic sediment impacts on different coral and symbiont tolerance thresholds impacted by dredging activity [24,63].

No mortality or significant tissue loss was observed in *M. cavernosa* or *S. intersepta* at the suspended sediment concentrations used in the study. This is contrary to the meta-analysis conducted by Tuttle and Donahue that reported mortality of adult corals can occur in suspended sediment in concentrations as low as 3.2 mg L^−1^ with the lowest-observed adverse-effect levels ranging from 10-100 mg L^−1^ on adult corals [31]. Some threshold values for Total Suspended Solids (TSS) range from as little as 3.3 to 260 mg L^−1^, which is lower than the 511.7 mg L^−1^ found to have growth effects on *M. cavernosa* and *S. intersepta* by day 30 in this study [14]. These wide ranges of values may be reflective of species sensitivity, morphology, exposure duration and sediment grain size or other physical characteristic [14,31,64]. Sediment grain size may also influence the effects of deposited sediment on coral, but data have been variable. Clay- and silt-sized sediment is thought to be removed more easily than sand by some species, however, these fine sediments may also harbor pollutants that can be harmful to coral [14,64]. The sand content of the sediment used here was close to the fraction seen in Australian offshore environments near reef habitats after dredging activity, therefore, similar responses from grain-size could be assumed between our *ex situ* data and *in situ* field responses [24].

Though not measured, there was evidence of sediment settling. As turbidity declined in the chambers of the higher sediment treatments (142.1 mg L^−1^ and higher) sediment was observed on coral surfaces of both *M. cavernosa* and *S. intersepta* (Supplementary Figure 13). Additionally, the temperature loggers in the sediment chambers had accumulated layer sediment indicating that some sediment deposition occurred (Supplementary Figure 14). This suggests that *M. cavernosa* and *S. intersepta* can remove sediment rapidly enough to prevent tissue loss with short-term exposures. Most laboratory experiments have utilized small fragments of an adult coral colony. Although the use of small fragments is to conserve a valuable resource, these small fragments do not adequately represent the response of a whole coral colony whose morphology may be instrumental in its responses [28,31]. Flores indicated that coral mortality of 5 cm diameter fragments was related to sediment deposition on the coral surface and presented with exposures [65]. The absence of mortality or tissue loss in this study may be due to the morphology of the test colonies or the sediment-rejection abilities of these coral species [66].

Sediment generated by dredging operations impacts coral reefs [14,17–20]. One pertinent example of significant impacts of dredging on coral occurred during the 2013-2015 dredging of the Port Miami. Corals were likely affected up 10 km from the dredge site due to the movement of suspended sediments, underscoring the need for a better understanding to prevent coral loss [67]. This study is the first to provide details of water column suspended sediment on Atlantic/Caribbean coral species that are needed for more standardized reporting and establishing threshold values [31]. It is essential to understand that the turbidity values in this study were not consistent throughout the exposure period. To achieve targeted values, pumps in the experimental chambers were used to resuspend sediment twice per day. *In situ* exposure to continuous suspended sediment may result in tissue death or mortality, which were not observed in this study. The results from this project highlight the tolerance of *M. cavernosa* and *S. intersepta* in response to short-term, pulsed exposures (10-day) to mean turbidity levels at or below 198 NTU. Longer-term, pulsed exposures (30-day) at 347 NTU do not alter bleaching indicators, but calcification is affected in *M. cavernosa* and *S. intersepta*. The lack of coral tissue mortality provides evidence that at least some Atlantic coral species are tolerant to sediment pulses in a longer-term (30 day) exposure. If coral can remove sediment, there is evidence that it is able to recover from sediment induced stress [28]. Overall, the results of this study indicate that *M. cavernosa* and *S. intersepta* could recover from pulse generated dredging events, assuming no additional stressors are present.

## CONCLUSION

To effectively manage dredging operations in coral reef habitats, considerations must be made in cause-effect pathways for coral responses. This study is the first to report on all three water quality parameters associated with cause-effect pathways in a laboratory setting and is a measured step towards the standardization of reporting multiple water quality metrics to better inform assessments. The coral response data presented in this study will assist regulators and managers to better understand and calculate the significant impacts of dredging in coral reef habitats. Additional research is fundamental for better predictive impacts and should include more and varied coral species, water current influences, spatial and temporal dynamics of a reef habitat and other local and global stressors that may act synergistically with sediment.

## Supplementary Material

Supplement1

## Figures and Tables

**Table 1:** Mean and standard deviation values for sediment related water quality metrics for comparisons and for exposure days 0-10 and 0-30.

		Comparisons at Day 0			Day 0-10	Day 0-30	
Dose: Dry weight by vol (mg L^−1^)	Dose: Dry weight by surface area (mg cm^−2^) of chamber	Turbidity (NTU) n=4	PAR reduction (%) n=4	TSS (mg L^−1^) n=4	Turbidity (NTU) n=28	Turbidity (NTU) n=76	PAR reduction (%) n=20
0	0.88	0.54 (0.14)	0.00 (0.00)	11.50 (10.97)	0.87 (0.06)	1.15 (1.03)	0.00 (0.00)
5.4	1.75	38.50 (1.88)	44.52 (5.75)	64.00 (5.89)	8.67 (12.02)	11.88 (14.12)	46.85 (9.39)
73.9	3.5	79.32 (10.35)	57.34 (4.35)	107.50 (7.33)	18.45 (28.05)	26.02 (31.15)	56.78 (14.05)
142.1	7	163.75 (9.71)	75.13 (3.00)	237.00 (29.90)	37.80 (53.90)	60.41 (67.24)	70.19 (12.15)
284.3	32	319.00 (52.60)	84.64 (1.93)	379.20 (42.00)	77.70 (107.00)	138.00 (132.80)	81.11 (7.39)
511.7	80	558.80 (34.80)	92.49 (2.21)	695.20 (30.00)	181.00 (222.20)	303.70 (246.30)	91.12 (3.83)

**Abbreviations:** PAR-Photosynthetically Active Radiation; NTU-Nephelometric Turbidity Unit; TSS-Total Suspended Solids.
