# Supplementary material for: Effects of Dredging Induced Resuspension of Fine-Grain Sediment on Two Scleractinian Corals, Montastraea cavernosa and Stephanocoenia intersepta
Source: J Coast Zone Manag. Author manuscript; Available in PMC 2026 Aug 1. (PMC13426912)
Supplement: Supplement1 [file NIHMS2079436-supplement-Supplement1.docx]

Supplementary Material

Effects of dredging induced resuspension of fine-grain sediment on stony corals, *Montastraea cavernosa* and *Stephanocoenia intersepta*

Hankins, C.*, Bahr, K., White, D., Jones, Y., Glahn, A., Lehmann, Kosmynin, V., Fisher, W.S.

*** Correspondence:** [Hankins.cheryl@epa.gov](mailto:Hankins.cheryl@epa.gov)

# Supplementary Figures and Tables


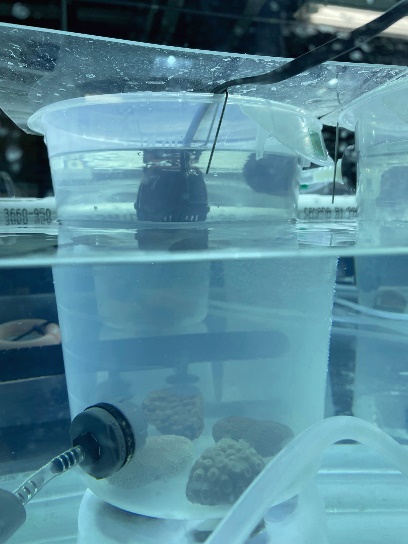


(C)

(B)

(A)

**Supplementary Figure 1.** Experimental chamber setup containing: (A) two coral colonies from each species *Montastrea cavernosa* and *Stephanocoenia intersepta,* (B) stainless steel wire hanger, and (C) circulation pump.


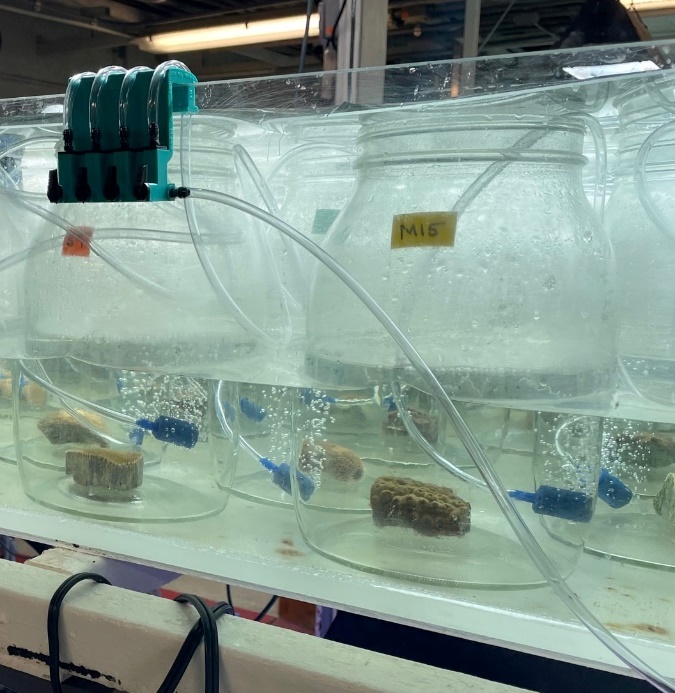


**Supplementary Figure 2.** Individual coral colonies in 3.7 L glass containers during 24-hr incubation time between Total Alkalinity measurements.

**Supplementary Figure 3.** Mean alkalinity levels at days 0-10 and days 0-30. Error bars represent standard deviation of the mean response for each treatment.

**Supplementary Figure 4.** Mean magnesium levels at days 0-10 and days 0-30. Error bars represent standard deviation of the mean response for each treatment.


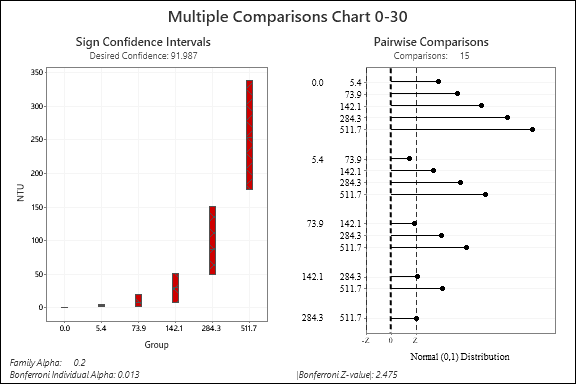

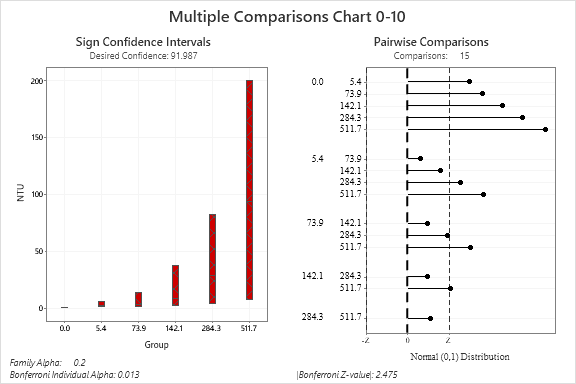


(B)

(A)

**Supplementary Figure 5.** Multiple comparison charts for mean turbidity (NTU) for exposure days (A) 0-10 (Bonferroni, comparisons = 15, Ties = 7, ɑ = 0. 2, Z = 2.475) and (B) 0-30 (Bonferroni, comparisons = 15, Ties = 57, ɑ = 0. 2, Z = 2.475). Vertical, bold dashed line is a baseline, the vertical, Z dashed lines are confidence intervals. Solid, horizontal lines extending beyond the Z line indicates a significant difference between the two treatments listed.

**Supplementary Table 1.** Mean turbidity (NTU) values and standard deviation (SD) for pre- and post-stir measurements and percent reduction for each treatment exposure days 0-30.

|  | NTU (SD) | |  |
| --- | --- | --- | --- |
| Treatment (mg L^-1^) | Pre-stir | Post-sitr | Reduction (%) |
| 0 | 1.09 (1.31) | 1.23 (0.73) | 11.6 |
| 5.4 | 1.60 (1.14) | 23.63 (13.34) | 93.2 |
| 73.9 | 4.13 (6.64) | 50.39 (28.78) | 91.8 |
| 142.1 | 10.54 (16.76) | 117.59 (57.12) | 91.0 |
| 284.3 | 52.50 (71.00) | 239.90 (113.30) | 78.1 |
| 511.7 | 132.80 (149.60) | 493.6 (165.70) | 73.1 |


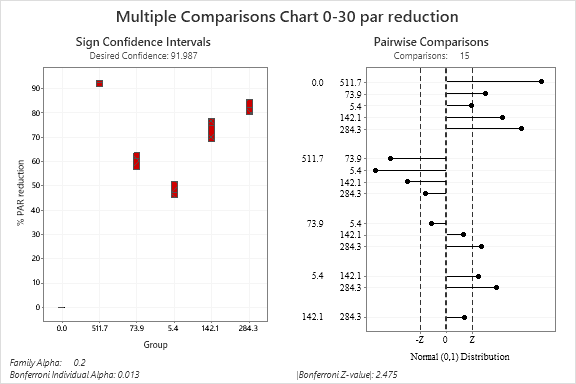

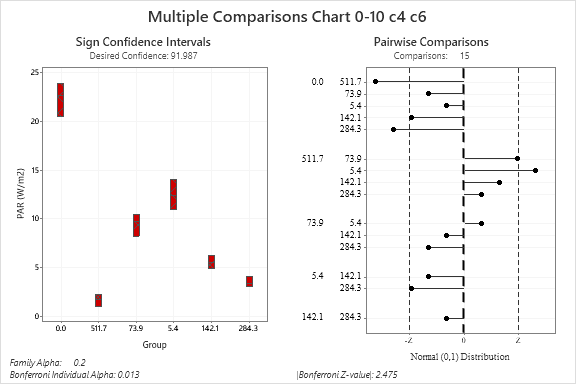


(B)

(A)

**Supplementary Figure 6.** Multiple comparisons chart for mean reduction of Photosynthetically Active Radiation at (A) 0-10 day exposure period (Bonferroni, comparisons = 15, ɑ = 0. 2, Z = 2.475) and (B) 0-30 day exposure period (Bonferroni, comparisons = 15, ties = 114. ɑ = 0.2, Z = 2.475). Vertical, bold dashed line is a baseline, the vertical, Z dashed lines are confidence intervals. Solid, horizontal lines extending beyond the Z line indicates a significant difference between the two treatments listed.

**Supplementary Figure 7.** Mean zooxanthellae density normalized to tissue surface area for *Montastraea cavernosa* and *Stephanocoenia intersepta* at day 10. Error bars represent standard error of the mean response of species for each treatment.

**Supplementary Figure 8.** Mean zooxanthellae density normalized to tissue surface area for *Montastraea cavernosa* and *Stephanocoenia intersepta* at day 30. Error bars represent standard error of the mean response of species for each treatment.

**Supplementary Figure 9.** Mean chlorophyll-*a* concentration normalized to tissue surface area for *Montastraea cavernosa* and *Stephanocoenia intersepta* at day 10. Error bars represent standard error of the mean response of species for each treatment.

**Supplementary Figure 10.** Mean chlorophyll-*a* concentration normalized to tissue surface area for *Montastraea cavernosa* and *Stephanocoenia intersepta* at day 30. Error bars represent standard error of the mean response of species for each treatment.

**Supplementary Figure 11.** Mean protein concentration normalized to tissue surface area for *Montastraea cavernosa* and *Stephanocoenia intersepta* at day 10. Error bars represent standard error of the mean response of species for each treatment.

**Supplementary Figure 12.** Mean protein concentration normalized to tissue surface area for *Montastraea cavernosa* and *Stephanocoenia intersepta* at day 30. Error bars represent standard error of the mean response of species for each treatment.


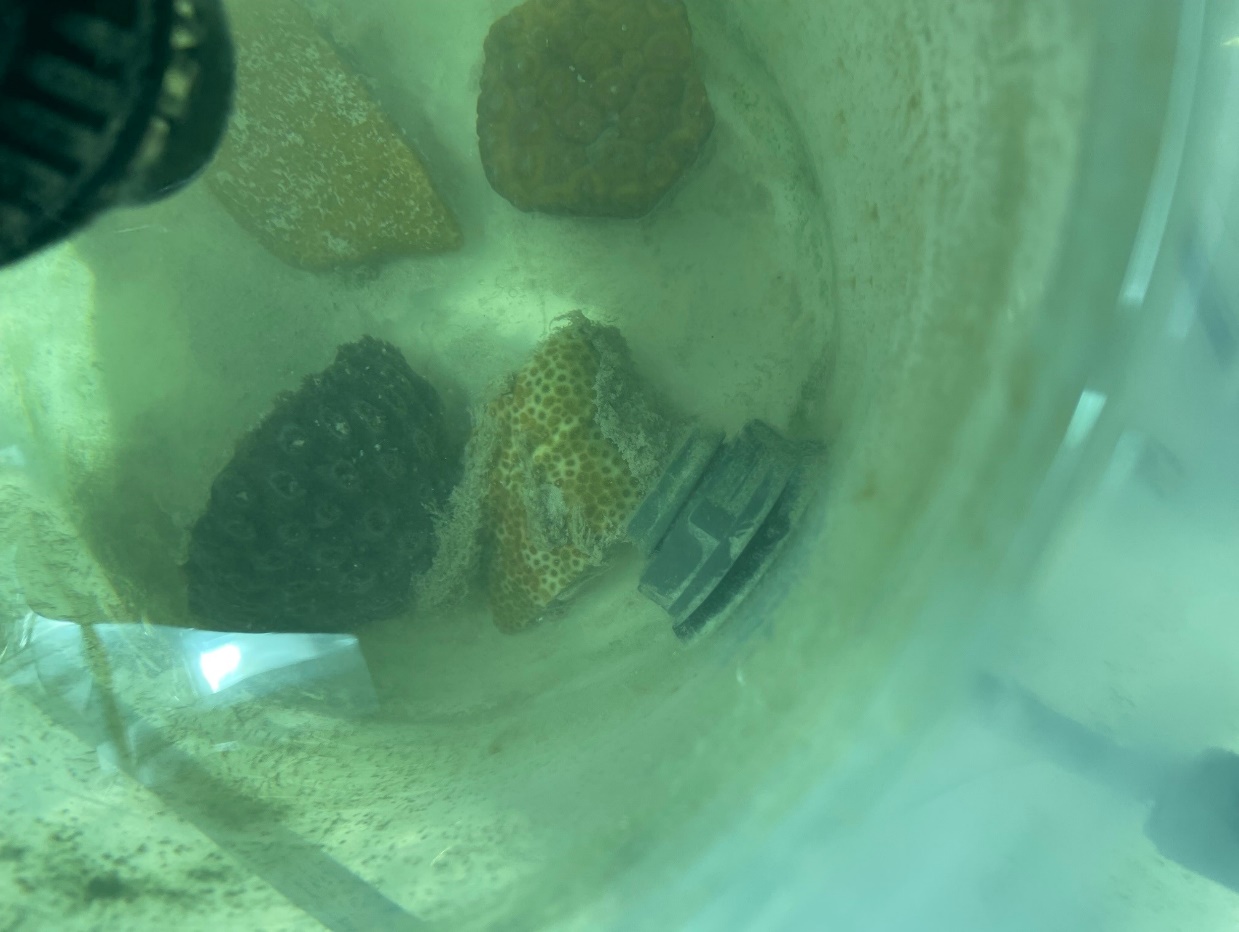


Sediment

**Supplementary Figure 13**. Top-down view of an experimental chamber in the 142.1 mg L^-1^ treatment prior to resuspending sediment. *Montastraea cavernosa* colonies are in the top right and bottom left of image, *Stephanocoenia intersepta* colonies are in the top left and bottom right of image. A few areas with sediment have been indicated with arrows.


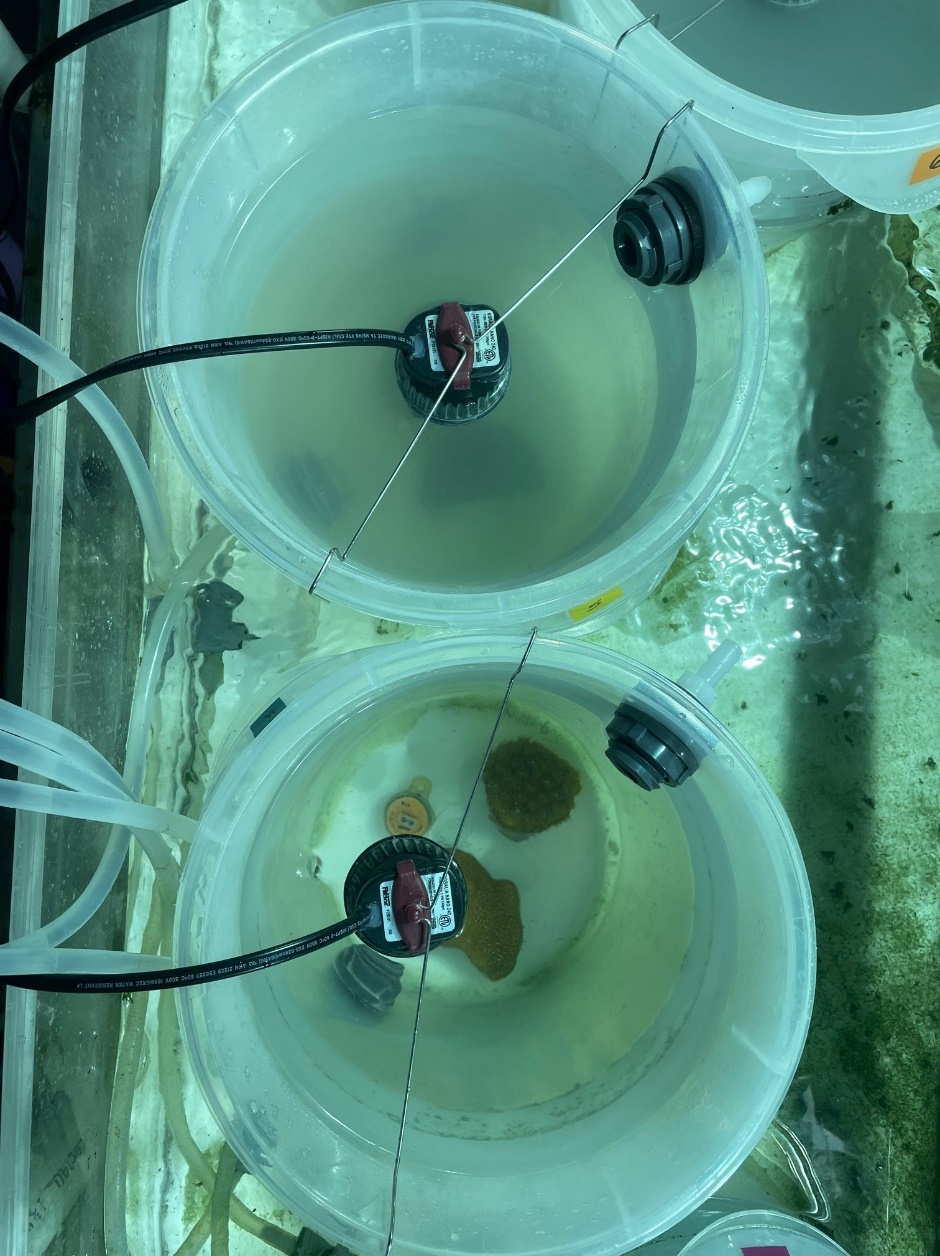


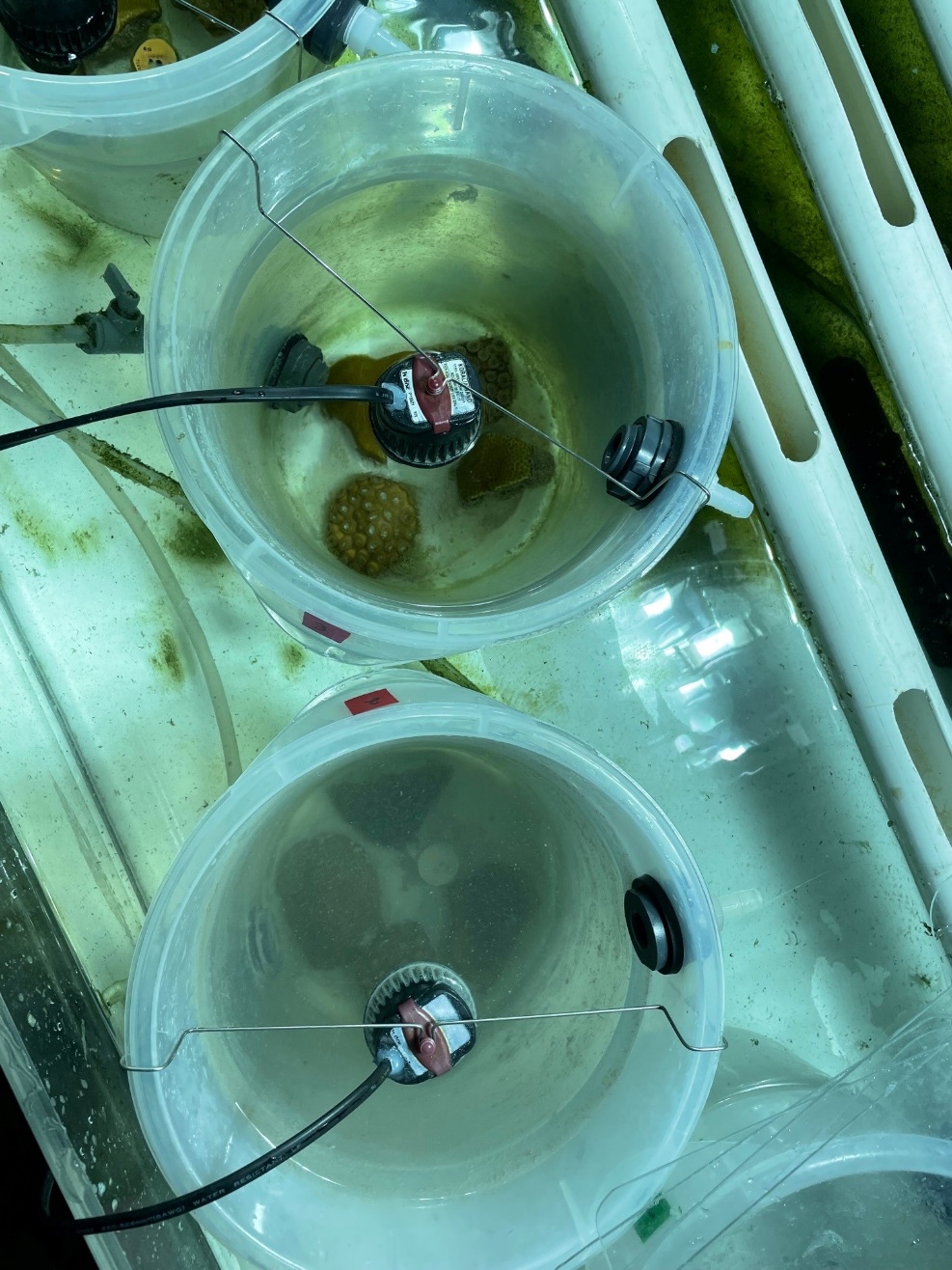


(B)

(A)

**Supplementary Figure 14.** Images of temperature loggers (circled). (A) Image of logger with no sediment accumulation and (B) image of logger with accumulated sediment.
